# Comparative Analysis of the Enzymatic, Coagulant, and Neuromuscular Activities of Two Variants of *Crotalus durissus ruruima* Venom and Antivenom Efficacy

**DOI:** 10.3390/ph18010054

**Published:** 2025-01-06

**Authors:** Poliana J. Demico, Isabele N. Oliveira, Vitória S. Proença-Hirata, Samuel R. Dias, Hugo A. Ghirotti, Elisangela O. Silva, Inês C. Giometti, Francis L. Pacagnelli, Kristian A. Torres-Bonilla, Stephen Hyslop, Nathália C. Galizio, Karen de Morais-Zani, Manuela B. Pucca, Anderson M. Rocha, Jéssica B. Maciel, Marco A. Sartim, Wuelton M. Monteiro, Rafael S. Floriano

**Affiliations:** 1 Laboratory of Toxinology and Cardiovascular Research, University of Western São Paulo (UNOESTE), Presidente Prudente 19050-680, SP, Brazil; polidemico@gmail.com (P.J.D.); isabelebiomed@outlook.com (I.N.O.); vicproenca.vp@gmail.com (V.S.P.-H.); samueldias1999@hotmail.com (S.R.D.); hugoghirotti@hotmail.com (H.A.G.); elisangela@unoeste.br (E.O.S.); inesgiometti@unoeste.br (I.C.G.); francispacagnelli@unoeste.br (F.L.P.); 2 Department of Pharmacology, Faculty of Medical Sciences, State University of Campinas (UNICAMP), Campinas 13083-888, SP, Brazil; kristiantorres19@gmail.com (K.A.T.-B.); hyslop@unicamp.br (S.H.); 3 Laboratory of Pathophysiology, Butantan Institute, São Paulo 05585-000, SP, Brazil; nati_galizio@hotmail.com (N.C.G.); karen.zani@butantan.gov.br (K.d.M.-Z.); 4 Department of Clinical Analysis, School of Pharmaceutical Sciences, São Paulo State University (UNESP), Araraquara 14800-903, SP, Brazil; manupucca@hotmail.com; 5 Graduate Program in Tropical Medicine, State University of Amazonas (UEA), Manaus 69850-000, AM, Brazil; amr.0609@gmail.com (A.M.R.); jessicabmaciel@hotmail.com (J.B.M.); marcosartim@hotmail.com (M.A.S.); 6 Department of Research and Development, Nilton Lins Foundation, Manaus 69058-030, AM, Brazil

**Keywords:** *Crotalus durissus* venom, coagulant activity, enzymatic activity, intraspecific variation, neurotoxicity, neutralization by antivenom

## Abstract

**Background:** We compared the enzymatic, coagulant, and neuromuscular activities of two variants (yellow—CDRy and white—CDRw) of *Crotalus durissus ruruima* venom with a sample of *C. d. terrificus* (CDT) venom and examined their neutralization by antivenom against CDT venom. **Methods:** The venoms were screened for enzymatic and coagulant activities using standard assays, and electrophoretic profiles were compared by SDS-PAGE. Neutralization was assessed by preincubating venoms with crotalic antivenom and assaying the residual activity. **Results:** SDS-PAGE showed that the venoms had similar electrophoretic profiles, with the main bands being phospholipase A_2_ (PLA_2_), serine proteinases, L-amino acid oxidase (LAAO), and phosphodiesterase. CDRy venom had the highest proteolytic and LAAO activities, CDRw venom had greater PLA_2_ and esterolytic activities at the highest quantity tested, and CDT had greater PLA_2_ activity than CDRy. CDRw and CDT venoms had similar proteolytic and LAAO activities, and CDRy and CDT venoms had comparable esterolytic activity. None of the venoms altered the prothrombin time (PT), but all of them decreased the activated partial thromboplastin time (aPPT); this activity was neutralized by antivenom. The minimum coagulant dose potency was CDRw >> CDRy > CDT. All venoms had thrombin-like activity that was attenuated by antivenom. CDRy and CDRw venoms showed α-fibrinogenolytic activity. All venoms partially cleaved the β-chain. CDRy and CDT venoms caused neuromuscular facilitation (enhanced muscle contractions) followed by complete blockade, whereas CDRw venom caused only blockade. Antivenom neutralized the neuromuscular activity to varying degrees. **Conclusions:** These findings indicate that while CDR and CDT venoms share similarities, they also differ in some enzymatic and biological activities and in neutralization by antivenom. Some of these differences could influence the clinical manifestations of envenomation by *C. d. ruruima* and their neutralization by the currently used therapeutic antivenom.

## 1. Introduction

In Latin America, envenomations by Viperidae snakes are a serious public health problem [1,2,3]. In Brazil, these snakes are represented by three main genera, i.e., *Bothrops*, *Crotalus,* and *Lachesis* (Viperidae: Crotalinae), that, together, are responsible for more than 20,000 snakebites per year, as reported by the Notifiable Diseases Information System of the Brazilian Health Ministry (Brasília, DF, Brazil) [4]. The genus *Crotalus* includes ~50 species and subspecies of rattlesnakes widely found throughout the Americas [5,6]. In Brazil, this genus is exclusively represented by a single species (*Crotalus durissus*), with four subspecies (*C. d. durissus*, *C. d. marajoensis*, *C. d. ruruima,* and *C. d. terrificus*). Recently, the subspecies *C. d. cascavella* and *C. d. collilineatus* were synonymized with *C. d. terrificus* due to a considerable overlap in their geographic distributions [7].

Envenomation by *C. durissus* is frequently characterized by severe systemic clinical manifestations such as rhabdomyolysis, acute renal failure, coagulopathy, and neuromuscular paralysis [8,9,10,11,12]. These effects are mostly mediated by phospholipase A_2_ (PLA_2_, as part of the crotoxin complex), with contributions from C-type lectin-like proteins (CTL) and snake venom serine proteases (SVSPs) [5,8,10,13,14,15,16]. Crotoxin (a heterodimeric PLA_2_ presynaptically active β-neurotoxin), the most abundant and most active toxin in these venoms, is responsible for peripheral neuromuscular paralysis that results in respiratory impairment [5,17,18,19] and also contributes to rhabdomyolysis [9,18,20,21,22,23,24] and renal failure [18,25,26]. In addition, SVSPs present in these venoms exert thrombin-like activity that results in coagulation disturbances [27,28]. These venoms also contain crotamine and convulxin that contribute to peripheral and central neurological dysfunctions [29,30,31] and platelet disorders [31,32]. Crotamine has a variable expression among specimens of *C. durissus* and is a well-known example of intraspecific variation in these venoms [33,34].

*Crotalus d. ruruima* is found in the northern Brazilian Amazon state of Roraima [35,36] and is geographically isolated from *C. d. terrificus* that occurs outside the Amazon region, i.e., in northeastern, central, southeastern, and southern Brazil [37]. Envenomation by *C. d. ruruima* produces local effects such as pain, edema, bleeding, eruptions, and necrosis, as well as systemic alterations characterized by potent neurotoxicity reflected as blurred vision, muscle weakness, myasthenic facies, ophthalmoplegia, rhabdomyolysis, and acute kidney injury [5,8,10,14,15,16,38]. The clinical treatment for envenomation by *C. durissus* subspecies (ssp.) in Brazil involves the use of therapeutic equine antivenom (AV) raised against *C. d. terrificus* venom [8,38].

Compared to other *C. durissus* ssp., such as *C. d. terrificus*, the basic toxinology of *C. d. ruruima* remains poorly understood, and only in recent years has it become the focus of more detailed studies (see [8,38] and references therein). Dos Santos et al. (1993) [39] provided one of the earliest characterizations of *C. d. ruruima* venom and its two color variants (yellow and white). White venom was more lethal to mice than yellow venom when administered intramuscularly and intraperitoneally, but both showed similar coagulant activity, were hemolytic, and caused moderate mouse paw edema. Yellow venom was approximately fourfold more proteolytic than white venom and caused hemorrhage and mild necrosis in mice; yellow venom contained crotamine. The biological activities were neutralized to varying degrees by crotalic and bothropic-crotalic antivenoms. A subsequent report [40] described individual variability in the lethal, PLA_2_, coagulant, myonecrotic, and hemorrhagic activities of these venom variants. Proteomic analysis of *C. d. ruruima* venom revealed the presence of toxin families common to other *C. durissus* ssp. (but in different proportions), including crotoxin (which accounted for ~83% of the venom protein), CTLs, P-III class snake venom metalloproteinases (SVMPs), SVSPs, crotamine, and a low content of L-amino acid oxidase (LAAO) [5]. To date, the only *C. d. ruruima* venom components extensively characterized are PLA_2_ of the crotoxin complex that cause cytotoxicity, myonecrosis, edema, and platelet aggregation in vitro and neuromuscular blockade in vitro [41,42,43,44,45].

As shown by the foregoing discussion, although *C. d. ruruima* venom has been studied in some analyses of *C. durissus* ssp. venoms, few investigations have focused specifically on the basic toxinology of this subspecies. To expand our knowledge of venom from this subspecies, in this work, we compared the toxicological features of the yellow and white variants of *C. d. ruruima* venom with regard to their enzymatic, coagulant, and neuromuscular activities in vitro. Considering the geographical distribution of *C. d. ruruima* and *C. d. terrificus*, as mentioned above, we also assessed the efficacy of therapeutic crotalic AV in neutralizing the coagulant and neuromuscular activities of the venoms.

## 2. Results

### 2.1. Electrophoretic (SDS-PAGE) Profiles of the Venoms

**Figure 1:**
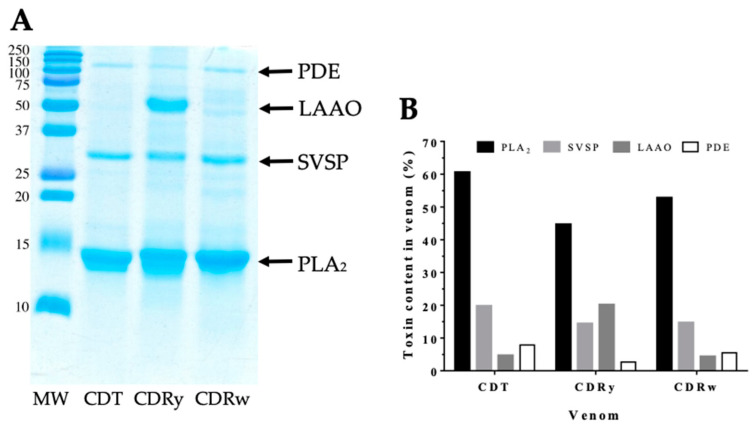
(**A**). Electrophoretic profiles of *C. d. terrificus* and *C. d. ruruima* venoms. Venom samples (20 µg) were applied to a 15% polyacrylamide gel and run by SDS-PAGE under reducing conditions, and the protein bands were then stained with Coomassie brilliant blue G-250. CDT—*C. d. terrificus*, CDRy—*C. d. ruruima* yellow venom, CDRw—*C. d. ruruima* white venom, MW—molecular weight markers (kDa). (**B**). Venom content of selected toxins based on densitometric analysis of the electrophoretic profiles. The toxin content was expressed as a percentage of the total densitometric analysis, and toxins were identified based on the molecular masses of the protein bands, as described in previous studies [13,46].

SDS-PAGE was used to examine the general profile and protein composition of the venoms. Figure 1A shows the SDS-PAGE electrophoretic profiles of CDT, CDRy, and CDRw venoms under reducing conditions. Overall, the electrophoretic profiles of the venoms were similar (except for a prominent ~50 kDa band present in CDRy venom) and displayed a profile typical of South American rattlesnakes. Based on their molecular masses, the principal protein bands in the range of 14–150 kDa were assigned to the main toxin families known to occur in *C. durissus* ssp. venoms, namely PLA_2_ (~14 kDa), SVSP (~30 kDa), LAAO (~50 kDa), and phosphodiesterase (PDE, ~100 kDa) [13,46]. Densitometric analysis showed that PLA_2_ was the most abundant toxin class in the venoms (45–60%), followed by SVSP (14–20%), LAAO (4.4–4.7%, except for CDRy venom, in which LAAO accounted for ~20% of total proteins, in agreement with the yellow color of this venom and its high LAAO activity [47] (see also Section 2.2 below) and PDE (2.7–7.9%) (Figure 1B). All of the venoms analyzed were crotamine-negative, as indicated by the absence of an ~5-kDa protein band.

### 2.2. Enzymatic Activities of Crotalus durissus (CDT, CDRy, and CDRw) Venoms

**Figure 2:**
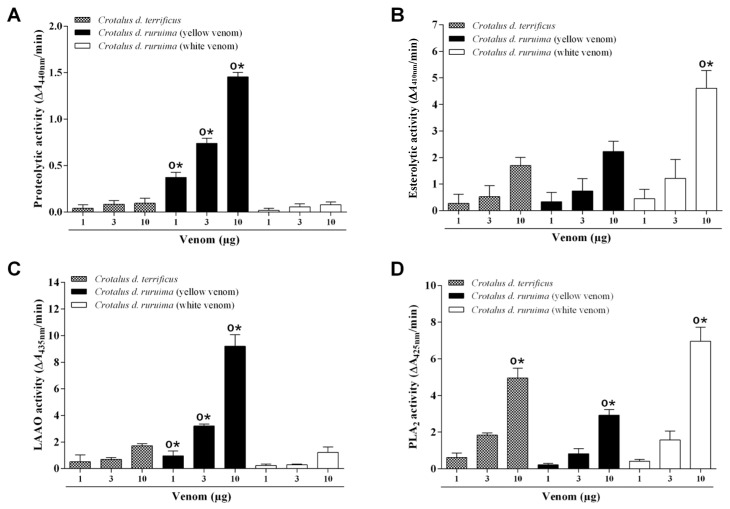
Proteolytic (**A**), esterolytic (**B**), L-amino acid oxidase (LAAO) (**C**), and PLA_2_ (**D**) activities of *C. d. terrificus* (CDT) and *C. d. ruruima* (CDRy and CDRw) venoms. The columns represent the mean ± SD (*n* = 3). * *p* < 0.05 indicates significant differences among the three amounts of venom in panels (**A**,**C**) and between 10 μg of venom and the other two amounts in panels (**B**,**D**). ^o^ *p* < 0.05 indicates significant differences among the venom types (CDRy, CDRw, and CDT) for the corresponding amounts of venom (one-way ANOVA followed by the Tukey test in all cases).

The enzymatic activities of the venoms were compared by assaying four activities (proteolytic, PLA_2_, esterolytic, and LAAO). Figure 2 shows these activities in the venoms examined. CDRy venom (1–10 μg) had much higher proteolytic and LAAO activities than CDT and CDRw venoms at all quantities tested, with these two activities being similar in the latter two venoms. At the highest amount tested (10 μg), CDRw venom had higher esterolytic and PLA_2_ activities than CDT and CDRy venoms, while the activities at lower amounts were similar to the latter two venoms. The esterolytic and PLA_2_ activities of CDRy and CDT venoms were similar, although the PLA_2_ activity of CDT at the highest amount of venom was greater than for CDRy (Figure 2D).

### 2.3. Neuromuscular Activity of C. durissus Venoms in Mouse PND Preparations

**Figure 3:**
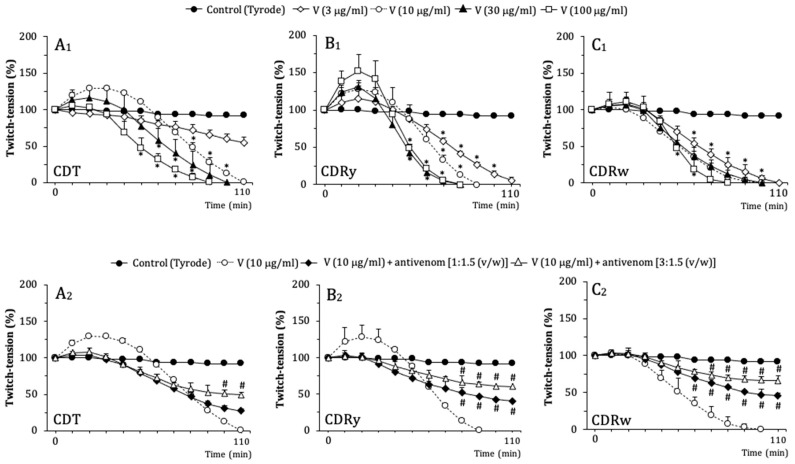
Neuromuscular activity of *C. durissus* venoms (CDT, CDRy, and CDRw) in mouse PND preparations and neutralization by crotalic AV. (**A_1_**,**A_2_**) CDT venom-induced neuromuscular facilitation followed by blockade (**A_1_**) and neutralization by AV at AV:venom ratios of 1:1.5 and 3:1.5 (*v*/*w*) (**A_2_**). (**B_1_**,**B_2_**) CDRy venom-induced neuromuscular facilitation followed by blockade (**B_1_**) and neutralization by AV at AV:venom ratios of 1:1.5 and 3:1.5 (*v*/*w*) (**B_2_**). (**C_1_**,**C_2_**) CDRw venom-induced neuromuscular blockade (**C_1_**) and neutralization by AV at AV:venom ratios of 1:1.5 and 3:1.5 (*v*/*w*) (**C_2_**); note that CDRw did not produce initial neuromuscular facilitation. The points represent the mean ± SD (*n* = 3) of the twitch-tension responses expressed as a percentage of the pre-venom (basal) values (considered as 100%). * *p* < 0.05 compared to control (Tyrode solution) preparations, and ^#^ *p* < 0.05 compared to venom alone (10 μg/mL) (one-way ANOVA followed by the Tukey test in all cases).

The neuromuscular blocking activity of the venoms was compared using mouse PND preparations in vitro. Figure 3A_1_–C_1_ shows that all venoms (3–100 μg/mL) produced concentration-dependent neuromuscular blockade in PND preparations, with the venoms of CDT and CDRy also causing varying degrees of muscle facilitation (enhanced muscle contractions resulting from increased presynaptic neurotransmitter release or direct stimulation of skeletal muscle) prior to the onset of blockade. With the exception of the lowest concentration of CDT venom, all concentrations of the three venoms caused complete neuromuscular blockade within 2 h. For CDT venom, 10 μg/mL produced the greatest facilitation after 20–30 min [% increase in muscle twitch amplitude: 30.4 ± 4.2%, *p* < 0.05 compared to control preparations (Tyrode alone), *n* = 3] (Figure 3A_1_). With CDRy venom, the two highest concentrations (30 and 100 μg/mL) produced similar blockade, with all concentrations causing neuromuscular facilitation; the highest concentration (100 μg/mL) produced the greatest facilitation, with a peak at 20 min [% increase in muscle twitch amplitude: 52 ± 12.7%, *p* < 0.05 compared to control preparations (Tyrode alone), *n* = 3] (Figure 3B_1_). In contrast to the other venoms, CDRw venom caused little or no neuromuscular facilitation at the concentrations tested (Figure 3C_1_).

Table 1 shows the times for 50% and 90% blockade by these venoms. At the lowest concentrations (3 and 10 μg/mL), CDRy and CDRw venoms were generally more potent than CDT venom in causing 50% and 90% blockade. At higher concentrations (30 and 100 μg/mL), CDT venom caused 50% blockade after similar intervals compared to CDRy and CDRw but was somewhat less potent in causing 90% blockade.

### 2.4. Neutralization of the Neuromuscular Activity by AV

The ability of therapeutic antivenom to neutralize the neurotoxicity of the venoms was assessed in preincubation experiments. The preincubation of CDT and CDRy venoms (10 μg/mL) with crotalic AV (AV:venom ratio 1:1.5 and 3:1.5, *v*/*w*) abolished the neuromuscular facilitation caused by these venoms [amplitude of facilitation (%): 30.4 ± 4.2 (CDT venom) compared to 4.5 ± 1.1 (CDT + AV 1:1.5, *v*/*w*) and 8.9 ± 1.8 (CDT + AV 3:1.5, *v*/*w*), and 52 ± 12.7 (CDRy venom) compared to 3.6 ± 1.0 (CDRy + AV 1:1.5, *v*/*w*) and 1.8 ± 0.4 (CDRy + AV 3:1.5, *v*/*w*); *p* < 0.05 for responses with AV compared to venom alone, *n* = 3 each] (Figure 3A_2_,B_2_). In addition, AV (AV:venom ratios of 1:1.5 and 3:1.5, *v*/*w*) attenuated the neuromuscular blockade induced by all three venoms (10 μg/mL), as indicated by the increase in the times required for 50% blockade (Table 2). For all venoms, an AV:venom ratio of 3:1.5 (*v*/*w*) was only slightly more effective in neutralizing the neuromuscular blockade than the manufacturer’s recommended AV:venom ratio of 1:1.5 (*v*/*w*), with the activity of CDRw venom being neutralized slightly better than the other venoms (based on the extent of blockade seen at the end of the incubations) (Figure 3A_2_–C_2_).

### 2.5. Morphological Damage in Diaphragm Muscle Exposed to C. durissus Venoms and Neutralization by AV

**Figure 4:**
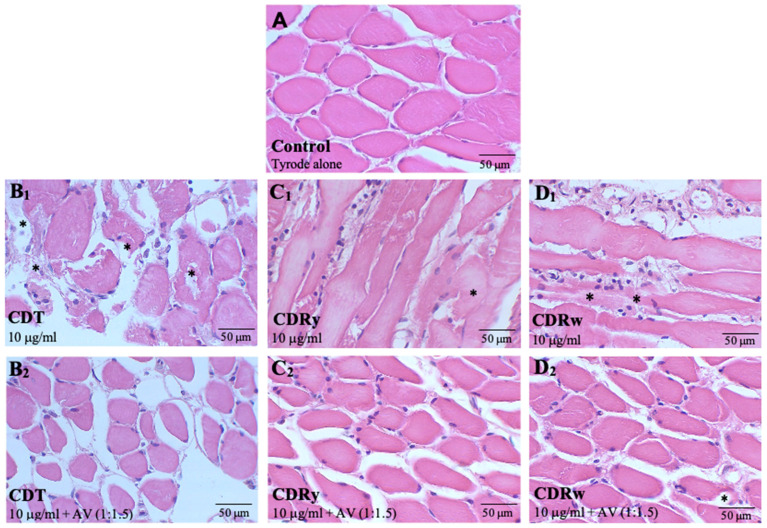
Histological alterations caused by *C. durissus* venoms (CDT, CDRy, and CDRw) in mouse PND preparations and their neutralization by crotalic AV. The preparations were incubated with Tyrode solution alone (control) (**A**), (**B_1_**,**B_2_**) CDT venom alone (**B_1_**) or pre-incubated with AV (**B_2_**), (**C_1_**,**C_2_**) CDRy venom alone (**C_1_**) or pre-incubated with AV (**C_2_**), and (**D_1_**,**D_2_**) CDRw venom alone (**D_1_**) or pre-incubated with AV (**D_2_**). Upon complete neuromuscular blockade or after 2 h (if complete blockade was not observed by this time), the preparations were processed for histological analysis. Myonecrosis (*) was the main histological alteration observed. Hematoxylin-eosin staining. Scale bars: 50 μm in all cases.

Histological analysis was used to assess the morphological damage caused by the venoms in diaphragm muscle. Figure 4 shows the morphological alterations in this muscle after incubation with the three venoms (10 μg/mL). Control preparations incubated with Tyrode solution alone for 2 h had a normal morphological appearance (undamaged muscle fibers with peripheral nuclei) (Figure 4A). All three venoms caused discrete-to-moderate myonecrosis after a 2 h incubation (Figure 4B_1_, Figure 4C_1_ and Figure 4D_1_ for CDT, CDRy and CDRw, respectively) that was prevented by preincubation with AV (AV:venom ratio of 1:1.5, *v*/*w*) (Figure 4B_2_, Figure 4C_2_ and Figure 4D_2_, respectively).

### 2.6. Coagulant Activity of C. durissus Venoms

The coagulant activity was assessed by examining the prothrombin and activated partial thromboplastin clotting times, the minimum coagulant dose, and the thrombin-like and fibrinogenolytic activities of the three venoms in order to allow a general comparison of their potencies, along with an assessment of their neutralization by AV.

#### 2.6.1. Effect of *C*. *durissus* Venoms on the Prothrombin (PT) and Activated Partial Thromboplastin (aPTT) Clotting Times and Neutralization by AV

**Figure 5:**
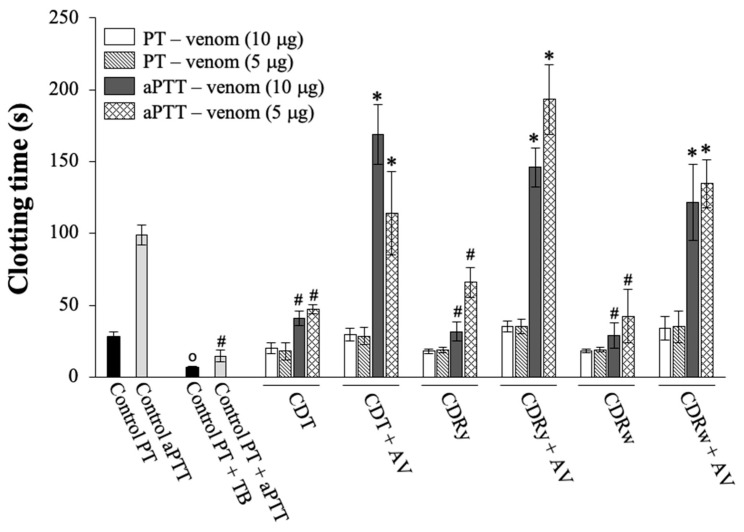
Coagulant activity of *C. durissus* venoms (CDT, CDRy, and CDRw) on the prothrombin (PT) and activated partial thromboplastin (aPTT) times in rat citrated plasma and their neutralization by crotalic antivenom (AV). The columns represent the mean ± SD (*n* = 4). * *p* < 0.05 compared to venom alone (CDT, CDRy, or CDRw, respectively) and ^o,#^ *p* < 0.05 compared to the PT (o) and aPTT (#) controls, respectively (One-way ANOVA followed by the Tukey test). TB—thrombin (2 U).

At the amounts tested (5 and 10 μg), none of the venoms affected the PT clotting time (control 28 ± 3.6 s vs. CDT 20.4 ± 3.8 s, CDRy 18 ± 1.6 s, and CDRw 18 ± 1.2 s for 10 μg of venom; *n* = 4 each) (Figure 5). In contrast, all of the venoms shortened the aPTT clotting time (control 99 ± 7 s vs. CDT 40.8 ± 5.1 s, CDRy 31.8 ± 6.9 s, and CDRw 28.8 ± 9 s for 10 μg of venom; *p* < 0.05 for all venoms compared to the control; *n* = 4 each). Preincubation with AV (AV:venom ratio of 1:1.5, *v*/*w*) completely prevented the decrease in aPTT clotting time caused by all three venoms (Figure 5).

#### 2.6.2. Minimum Coagulant Dose (MCD) of *C*. *durissus* Venoms

**Figure 6:**
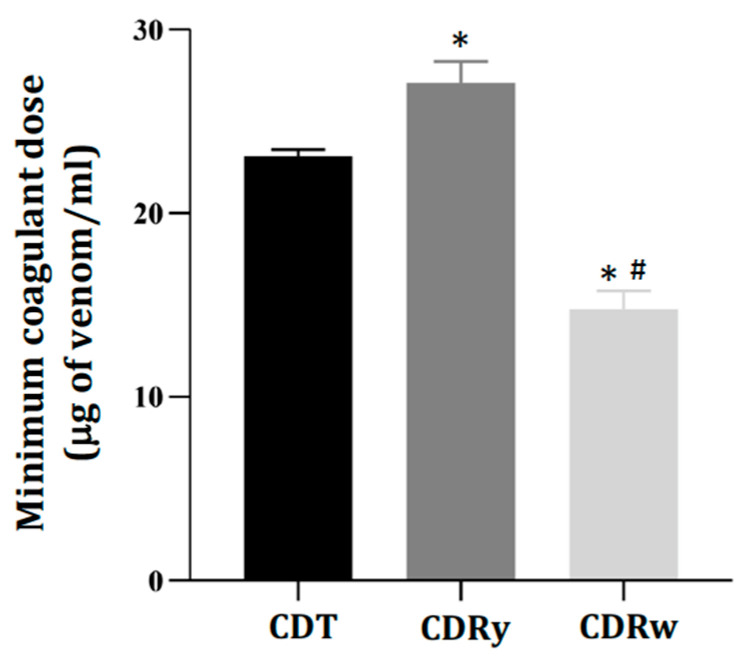
Minimum coagulant dose (MCD) of *C. durissus* venoms (CDT, CDRy, and CDRw) determined in human plasma. The columns represent the mean ± SD (*n* = 3). * *p* < 0.05 compared to CDT venom and ^#^ *p* < 0.05 compared to CDRy (one-way ANOVA followed by the Tukey test).

All of the venoms coagulated human citrated plasma, with CDRw venom being the most active [MCD (μg/mL): 14.8 ± 1, *p* < 0.05 compared to CDT and CDRy venoms, *n* = 3], followed by CDT [MCD (μg/mL): 23.1 ± 0.4, *n* = 3] and CDRy [MCD (μg/mL): 27.1 ± 1.2, *n* = 3] venoms (Figure 6). Both of the AV:venom ratios (1:1.5 and 3:1.5, *v/w*) tested effectively neutralized this coagulant activity and increased the coagulation time to >200 s (compared to the control time of ~60 s seen with the venoms alone), indicating effective neutralization. 

#### 2.6.3. Thrombin-like Activity of *C*. *durissus* Venoms and Neutralization by AV

**Figure 7:**
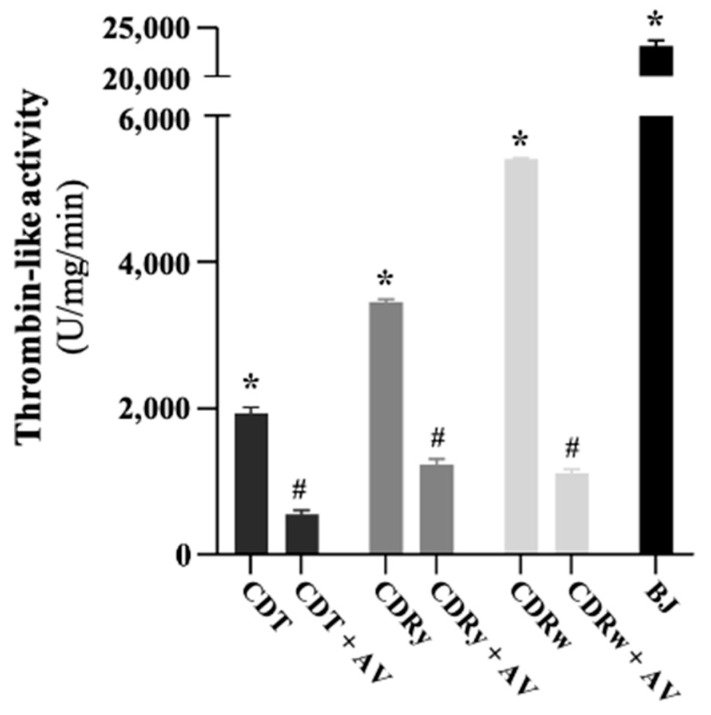
The thrombin-like activity of *C. durissus* venoms (CDT, CDRy, and CDRw) determined using the chromogenic substrate S-2238. The columns represent the mean ± SD (*n* = 3). Thrombin-like activity differed among the three venom types (CDT, CDRy, and CDRw; * *p* < 0.05) and was markedly attenuated by preincubation with crotalic AV (AV, 1:1.5, *v*/*w*) (^#^ *p* < 0.05 compared to the respective venom alone). BJ—*Bothrops jararaca* venom was used as a positive control and showed much greater activity (* *p* < 0.05) than the *Crotalus* ssp. venoms (one-way ANOVA followed by the Tukey test).

CDRw venom showed the highest thrombin-like activity [U/mg/min: 5413 ± 5.1, *n* = 3] when compared with CDRy and CDT venoms [U/mg/min: 3452 ± 32.7 (CDRy) and 1943 ± 75.8 (CDT), respectively, *n* = 3]. However, compared to *Bothrops jararaca* venom activity [U/mg/min: 23,159 ± 561, *n* = 3; positive control], the *Crotalus* venoms exhibited low thrombin-like activity towards the chromogenic substrate S-2238 (Figure 7). Preincubation with crotalic AV markedly inhibited this activity [residual activity, U/mg/min: 550 ± 50.3 (CDT; ~72% reduction), 1228 ± 87.5 (CDRy; ~64% reduction), and 1122 ± 45.6 (CDRw; ~80% reduction).

#### 2.6.4. Fibrinogenolytic Activity of *C*. *durissus* Venoms and Neutralization by AV

**Figure 8:**
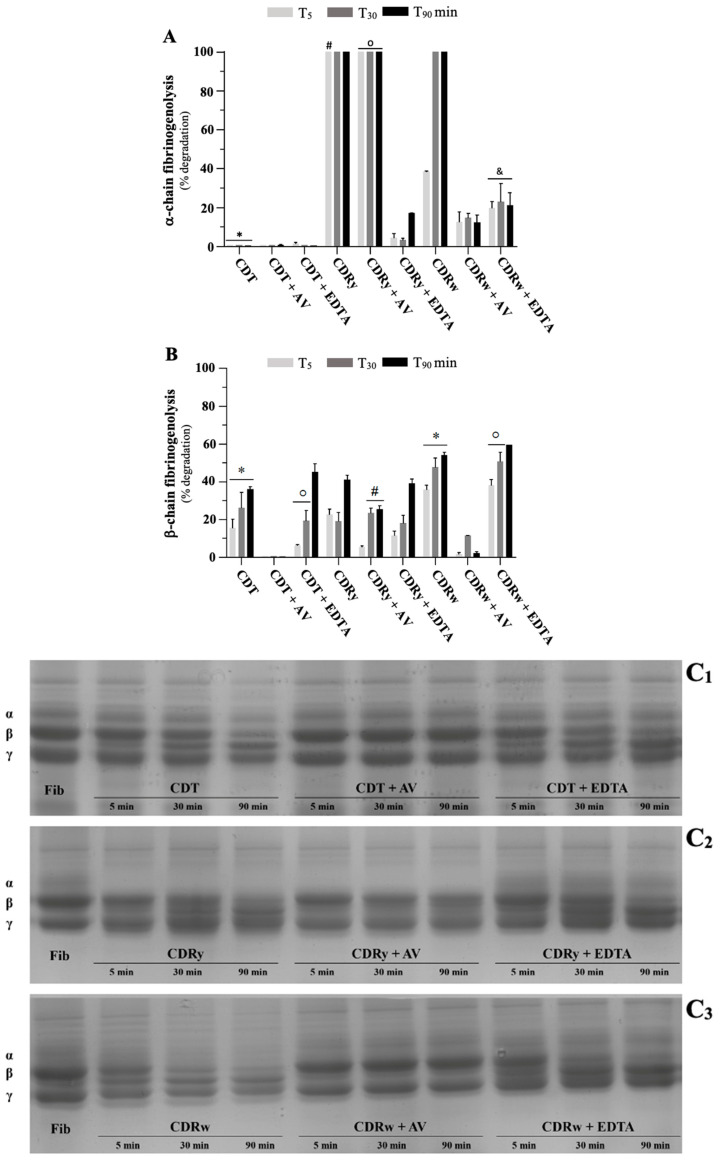
Fibrinogenolytic activity of *C. durissus* venoms (CDT, CDRy, and CDRw). Fibrinogen α-chain (**A**) and β-chain (**B**) degradation was assessed by SDS-PAGE after incubation with venoms for up to 90 min in the absence and presence of crotalic antivenom (AV) or EDTA. The activity, expressed as a percentage (%) of degradation, was estimated densitometrically, as described in Section 4.5.4. The columns represent the mean ± SD (*n* = 3). (**C_1_**–**C_3_**) SDS-PAGE electrophoretic profiles for degradation of the α-, β-, and γ-chains of fibrinogen after incubation with CDT (**C_1_**), CDRy (**C_2_**), and CDRw (**C_3_**) venoms for up to 90 min. In (**A**), * *p* < 0.05 compared to CDRy and CDRw venoms alone, ^#^ *p* < 0.05 compared to CDRw (5 min), ^o^ *p* < 0.05 compared to the corresponding time intervals for CDT + AV and CDRw + AV, and ^&^ *p* < 0.05 compared to CDT + EDTA and CDRy + EDTA. All assays were done using the same experimental conditions. In (**B**), * *p* < 0.05 indicates differences among the three-time intervals for the corresponding venoms, ^#^ *p* < 0.05 compared to the corresponding intervals for CDT + AV and CDRw + AV, and ^o^ *p* < 0.05 between the indicated time intervals for the corresponding venoms (one-way ANOVA followed by the Tukey test in all cases). All assays were done using the same experimental conditions.

Figure 8 shows the α- and β-fibrinogenolytic activities of CDT, CDRy, and CDRw venoms. Based on densitometric analysis using the γ-chain of fibrinogen as a reference (since its cleavage by snake venoms has not been described so far), CDT venom did not cleave the α-chain of fibrinogen (despite the α-chain band being visually less intense after a 90 min incubation; a lower intensity was also observed with the γ-chain). CDT venom gradually cleaved the β-chain, with some cleavage (15%) observed after 5 min of incubation. Preincubation of CDT venom with AV completely prevented cleavage of the β-chain, whereas preincubation with EDTA only partially inhibited this cleavage after 5 min of incubation.

In contrast to CDT venom, CDRy venom completely cleaved the α-chain after only 5 min of incubation (*p* < 0.05). The β-chain was cleaved gradually (but incompletely) from 5 min of incubation onwards. Preincubation with AV did not prevent cleavage of the α-chain and only partially inhibited cleavage of the β-chain (cleavage was 5–25% of that seen without AV). In contrast, EDTA prevented cleavage of the α-chain (cleavage was 3–17% of that seen without EDTA) but not the β-chain (cleavage was 11–39%, similar to venom alone), a finding similar to that observed for CDT venom. CDRw venom showed intense α- and β-fibrinogenolytic activity that degraded 100% and 54% of these chains, respectively, after 90 min. As observed for CDT venom, AV efficiently inhibited cleavage of the α- and β-chains of fibrinogen, while EDTA only partially inhibited cleavage of the α-chain.

## 3. Discussion

In this work, we examined the toxicological properties of two variants (CDRy and CDRw) of *C. d. ruruima* venoms and compared them with *C. d. terrificus* (CDT) to assess the possible influence of geographical variation on the venoms of these subspecies that are regularly involved in human envenomation [4]. We also assessed the efficacy of therapeutic crotalic AV produced by the Instituto Butantan in neutralizing the neurotoxic and coagulant activities of these venoms using in vitro experimental approaches.

Electrophoretic (SDS-PAGE) analysis of the venoms showed that the three venoms studied had similar overall profiles typical of *C. durissus* subspecies and contained the principal toxin groups or families known to occur in these venoms, i.e., PLA_2_, SVSP, LAAO, and CTL [5,13,46]. However, the venoms differed markedly in some of their enzymatic activities. CDRy had higher proteolytic and LAAO activities than CDRw and CDT venoms. This finding agreed with a previous study showing that CDRy venom had more proteolytic activity than CDRw and CDT venoms [39]. The yellow color of venoms reflects the presence of flavin derivatives associated with LAAO and generally results in high LAAO activity in such venoms [48,49], as also seen here with CDRw. CDRw also had higher esterolytic and PLA_2_ activities than CDRy and CDT venoms. This finding differs from that of Dos-Santos et al. (2005) [40], in which three samples of CDRw venom had less PLA_2_ activity than two samples of CDRy venom.

In PND preparations, CDT, CDRy, and CDRw venoms induced time- and concentration-dependent neuromuscular blockade. CDT venom (10–100 μg/mL) caused variable neuromuscular facilitation that was greatest at 10 μg/mL, followed by complete blockade during a 2 h incubation. The neuromuscular blockade caused by CDT venom (and *C. durissus* ssp. venoms in general) is mediated by crotoxin [41,50], a presynaptically active PLA_2_ β-neurotoxin directly responsible for the neurotoxicity and myotoxicity of these venoms [5], whereas facilitation is generally attributed to the small (4–5 kDa) myotoxic peptide crotamine [51,52] that modulates skeletal muscle voltage-gated potassium and sodium channel activity [30,53]. CDRy venom (3–100 μg/mL) showed intense neuromuscular facilitation followed by complete blockade caused by all concentrations within 2 h. In contrast, CDRw venom (3–100 μg/mL) caused no muscle facilitation but showed neuromuscular blockade that was generally more rapid in causing 90% blockade than CDT venom but less marked in relation to CDRy. The lack of neuromuscular facilitation by CDRw venom presumably allowed more rapid blockade with this venom in PND preparations. In agreement with this finding, other studies have shown that CDRw is more toxic than other varieties of CDR venoms [8,39,40,50]. These findings may indicate that envenomation by CDR could be potentially more severe for CDT. The neuromuscular blockade caused by CDT and CDR venoms resulted in moderate morphological changes in diaphragm muscle, with CDRw producing the most pronounced muscle changes, characterized mainly by myonecrosis. 

*Crotalus durissus* venom causes coagulopathy through the action of a thrombin-like component (procoagulant activity) and crotoxin (anticoagulant activity) [27,54,55,56]. The activities of both these components contribute to systemic hemorrhage during envenomation by *C. d. ruruima* [38]. Dos-Santos et al. (1993) [39] were the first to demonstrate that the venom of *C. d. ruruima* was more potent at inducing plasma coagulation than that of *C. d. terrificus*. Our findings corroborate this, showing that the yellow venom of *C. d. ruruima* had a lower minimum coagulant dose (MCD) than *C. d. terrificus* venom. In contrast, these authors found that yellow and white venoms of *C. d. ruruima* had similar potencies [39]. This finding differs from our results, which showed that CDRw venom had a lower MCD (was more potent) than CDRy. In a later study, Dos-Santos et al. (2005) [40] explored individual variations among yellow and white venoms of *C. d. ruruima* but found no consistent pattern for coagulant activity based on venom color. Overall, the coagulant activities observed here for CDT, CDRy, and CDRw venoms agree with the findings reported elsewhere [57], with *C. d. terrificus* venom showing the highest coagulant activity on human plasma compared to the subspecies *C. d. cascavella* and *C. d. collilineatus*. As shown elsewhere, procoagulant activity tends to be highly variable among *C. durissus* ssp., regardless of venom color, subspecies, or geographic origin [58].

Gyroxin, an SVSP with thrombin-like activity, is widely distributed in variable proportions among the venoms of *C. durissus* ssp.: in *C. d. ruruima* venom, gyroxin accounts for 8.1% of the venom, while in *C. d. terrificus,* the venom content of this toxin can reach 25.3%. Gyroxin is responsible for the procoagulant activity of *C. d. terrificus* venom [59,60], as demonstrated by Sousa et al. (2019) [60] and Barros et al. (2011) [61] by determining the MCD of isolated gyroxin. Interestingly, the CDT venom used in this work showed lower coagulant activity compared with CDRw venom, despite the former having a greater content of SVSP than the latter. This divergence may reflect a relatively higher abundance of SVMP in *C. d. ruruima* venom compared to CDT. In addition, the thrombin-like activity was higher in CDRw venom than in CDRy and CDT venoms, although this did not correlate closely with the estimated proportion of SVSP in these venoms. However, since the CDT sample studied here was obtained from a single specimen, it may not represent the coagulant profile of this subspecies since *C. d. terrificus* venoms show considerable individual variation [49,58,62,63]. Individual and populational variations in venom composition have already been described for *C. d. cumanensis* [64], *C. d. ruruima* [40], and *C. d. collilineatus* [58,65,66] venoms.

Snake venom proteases usually show specificity in their cleavage of the α- and β-chains of fibrinogen. SVMP actively cleave the α-chain of fibrinogen, while SVSP (with thrombin-like activity) preferentially cleave the β-chain [67]. Interestingly, CDRy and CDRw venoms were able to cleave the α-chain, although at different rates (CDRy completely degraded the α-chain after only 5 min of incubation, while CDRw degraded ~38% of the α-chain within a similar time scale). CDT venom, on the other hand, only cleaved the β-chain of fibrinogen. These results suggest that SVMP present in CDR venom, especially CDRy, cleave the α-chain of fibrinogen, a conclusion corroborated by the finding that EDTA inhibited the α-fibrinogenolytic activity of these venoms (CDRy and CDRw). Cleavage of the α-chain of fibrinogen has been extensively reported for venoms with a high content of SVMP, including those of the genera *Bothrops* [68] and *Lachesis* [69] and also for *C. d. cumanensis* [64,70], a subspecies found in Colombia and Venezuela that is known for its high content of SVMP [5]. In agreement with this inference regarding the presence of SVMP in CDR venoms, a previous proteomic study found that SVMP (exclusively P-III class) accounted for a low content (2.9%) of *C. d. ruruima* venom, similar to that of CDT venom (2.4%) in the same investigation [5], although the SVMP content in the latter venom can range from 0.09% to 5.5% [38].

Coagulopathy is a frequent finding in clinical envenomation by *C. durissus* in Brazil and primarily reflects fibrinogen consumption [27,54]. Coagulopathy is also a common finding in envenomation by *C. d. ruruima*, with non-clottable blood and hypofibrinogenemia occurring in 62.5% and 50% of reported cases, respectively, compared to <40% in envenomation by *C. d. terrificus* [38]. The epidemiological data and clinical findings reinforce the importance of studying the coagulant activities of different *C. durissus* subspecies, particularly since intraspecific variability can have a direct impact on the pathophysiology of envenomation.

Antivenom administration is the main therapeutic treatment for systemic envenomation by *Crotalus* spp. [71,72], with early administration being determinant in preventing or delaying clinical signs such as neuromuscular paralysis, respiratory impairment, rhabdomyolysis, acute renal failure, and coagulopathy [9,12,25,26]. In Brazil, the AV used to treat envenomation by *C. durissus* consists of F(ab’)2 fragments obtained by pepsin digestion of immunoglobulins raised in hyperimmunized horses using a pool of venoms that include two former subspecies of *C. durissus* (=*C. d. terrificus* and *C. d. collilineatus*) found in southeastern and southern regions of the country [71,73,74].

As shown here, AV neutralized the neuromuscular blockade caused by CDRy and CDRw venoms and also prevented the initial facilitatory effect induced by CDRy venom in mouse PND preparations. The AV was slightly more efficient in neutralizing the CDRw venom-induced neuromuscular blockade. Dos-Santos et al. (1993) [39] showed that this AV neutralized the hemolytic activity of CDRy and CDRw venoms in vitro and their lethality but was considerably less effective against CDT venom used to produce crotalic AV. Histological analysis showed that the AV attenuated the muscle damage caused by CDT and CDR venoms in mouse muscle diaphragm during the myographic experiments, especially that of CDRw venom, which showed the highest myotoxicity.

In the clotting tests, AV effectively inhibited the procoagulant activities of the three venoms on the intrinsic pathway (aPPT). The AV inhibited cleavage of the β-chain of fibrinogen but did not inhibit cleavage of the α-chain by CDRy venom. The efficacy of the crotalic AV produced by the Instituto Butantan in neutralizing the lethality of *C. durissus* spp. venoms is well-established, as this AV efficiently neutralizes the main toxins found in these venoms, including crotoxin (PLA_2_) and gyroxin (SVSP) [13]. Saravia et al. (2002) [75] have also reported the efficacy of crotalic AV in neutralizing the gyroxin-mediated procoagulant activity of *C. durissus* venoms, despite the fact that SVSP are usually poorly recognized by viperid antivenoms [76,77]. As shown here, the AV was unable to neutralize the α-fibrinogenolytic activity of CDRy venom. Since this activity is associated with the action of SVMP, this lack of neutralization may reflect the fact that the venom of this variant of *C. d. ruruima* is not used in the venom pool used to produce the AV, with the venoms actually used (*C. d. collilineatus* and *C. d. terrificus*) having a low content of SVMP.

This study has some limitations that could potentially influence the interpretation of the results and should be borne in mind. The primary limitation was the reduced number of specimens used to produce the venom pools, with venom from only one individual forming the CDT sample. Similarly, the number of geographic locations that provided venom samples was also limited, with venom from only one location forming the CDT sample. In view of the individual and geographic variation known to occur in *C. durissus* ssp. venoms, the low number of snakes and sampling sites suggests caution should be exercised in generalizing the findings reported here. An additional consideration relates to the procedures for venom collection, processing, and storage in different regions of the country and under different conditions. Although several factors related to this process can potentially affect venom properties, various studies with *Crotalus* spp. venoms have shown that the conditions of venom extraction, processing, and storage have only minor effects on venom characteristics such as electrophoretic and chromatographic profiles, enzymatic activities, and biological activities such as lethality, neurotoxicity, and hemodynamic effects [78,79,80,81,82,83]. These studies suggest it is unlikely that the conditions during venom extraction and storage markedly influenced the venom characteristics studied here.

## 4. Materials and Methods

### 4.1. Venoms and Antivenom

*Crotalus durissus ruruima* venoms were obtained from six adult snakes (four males and two females) captured in the Brazilian Amazon Forest in the vicinities of Boa Vista, Cantá, and Bonfim (state of Roraima); four specimens (three males and one female) produced white (CDRw) venom, and two specimens (one male and one female) produced yellow (CDRy) venom. *C. d. terrificus* (CDT) white venom was provided by the serpentarium of the Universidade do Vale do Paraíba (São José dos Campos, SP, Brazil) and consisted of a single extraction obtained from an adult female snake. The venoms were lyophilized and stored at −20 °C until used. All venom concentrations reported in this work refer to the dry venom weight and not to the venom protein content. When required, the venoms were weighed on an analytical balance and dissolved in ultrapure water or 0.9% saline solution and kept on ice until used. This study was registered with the Brazilian National System for the Management of Genetic Patrimony and Associated Traditional Knowledge (SISGEN, registration no. A7C040F).

Crotalic (anti-*Crotalus*) antivenom (AV) was obtained from the Instituto Butantan (São Paulo, SP, Brazil). This AV is raised in horses immunized with *C. d. terrificus* venom and consists of F(ab’)_2_ immunoglobulins [73,74]. According to the manufacturer’s specifications, 1 mL of AV neutralizes 1.5 mg of reference *C. d. terrificus* venom in a standard mouse lethality assay. The AV:venom ratios (expressed in volume:venom weight, *v*/*w*) used in the assays described below were based on this stated neutralizing capacity.

### 4.2. Animals

Male Unib:SW mice (25–30 g; 2–3 months old) obtained from the Multidisciplinary Center for Biological Investigation (CEMIB/UNICAMP) were transferred to the Experimental Animal House of the University of Western São Paulo (UNOESTE, Presidente Prudente, SP, Brazil) and housed in conventional polypropylene cages (5–10 mice/cage) with a wood-shaving substrate, at 23 ± 1 °C in ventilated stands (Alesco^®^, Hortolândia, SP, Brazil) on a 12-h light/dark cycle with lights on at 6 a.m. The mice had free access to food (Nuvital^®^, Colombo, PR, Brazil) and water. The animal experiments were approved by an institutional Committee for Ethics in Animal Use (CEUA/UNOESTE, protocol no. 7683/2023) and were done according to the general ethical guidelines for animal use established by the Brazilian Society of Laboratory Animal Science (SBCAL) and Brazilian legislation (Federal Law No. 11,794, of 8 October 2008), in conjunction with the guidelines for animal experiments established by the Brazilian National Council for Animal Experimentation (CONCEA) and The ARRIVE Guidelines 2.0 [84].

### 4.3. Sodium Dodecyl Sulphate-Polyacrylamide Gel Electrophoresis (SDS-PAGE)

Venom samples (20 mg diluted in sample buffer containing 2-mercaptoethanol) were applied to 15% polyacrylamide gels and run in SDS-PAGE [85], followed by staining with Coomassie brilliant blue G250 according to the manufacturer’s recommendations (GE Healthcare, Piscataway, NJ, USA). Molecular mass markers (Dual Color Precision Plus Protein Standards, BioRad, Carlsbad, CA, USA) were included in the run. The electrophoretic profiles were subsequently analyzed densitometrically using Gel Analyzer (version 23.1.1; developed by Istvan Lazar Jr. and Istvan Lazar Sr. and available at www.gelanalyzer.com; accessed on 9 October 2024).

### 4.4. Enzymatic Activities

#### 4.4.1. Phospholipase A_2_ (PLA_2_) Activity

Venom PLA_2_ activity was assayed essentially as described elsewhere [86]. The standard assay mixture contained 200 μL of buffer (10 mM Tris-HCl, 10 mM CaCl_2_, and 100 mM NaCl, pH 8.0), 20 μL of substrate (3 mM 4-nitro-3-octanoyloxy-benzoic acid) and 20 μL of venom (1 mg/mL solution prepared in ultrapure water) in a final volume of 240 μL. After adding the venom, the mixture was incubated for 30 min at 37 °C, and enzymatic activity was expressed as the initial velocity of reaction based on the increase in absorbance (425 nm) after 20 min. All assays were assayed in triplicate with readings at 30 s intervals using a SpectraMax 340 multiwell plate reader (Molecular Devices, Sunnyvale, CA, USA).

#### 4.4.2. Proteolytic (Caseinolytic) Activity

Proteolytic activity was assayed as previously described [87]. The standard assay mixture contained 90 μL of substrate (212 mM azocasein), 10 μL of reaction buffer (0.05 M Tris-HCl, 1 mM CaCl_2_, pH 8.0), and 10 μL of venom (1 mg/mL solution prepared in ultrapure water) in a final volume of 110 μL. The mixture was incubated for 90 min at 37 °C, and the reaction was then terminated by adding 200 μL of 5% trichloroacetic acid, followed by incubation for 5 min at room temperature, after which the mixture was centrifuged (8000× *g*, 5 min), and 150 μL of supernatant was transferred to a multiwell plate containing the same volume of 0.5 M NaOH. The resulting absorbance was read at 440 nm using a SpectraMax 340 multiwell plate reader (Molecular Devices), with one unit of activity being defined as an increase in absorbance of 0.001/min.

#### 4.4.3. Esterolytic Activity

Esterolytic activity was assayed essentially as described by Erlanger et al. (1961) [88] and adapted by Torres-Bonilla et al. (2020) [87]. The standard assay mixture contained 200 μL of substrate (100 mM Nα-benzoyl-DL-arginine 4-nitroanilide hydrochloride), 50 μL of reaction buffer (10 mM Tris-HCl, 10 mM CaCl_2_, 100 mM NaCl, pH 8.0), 15 μL of ultrapure water, and 5 μL of venom (1 mg/mL, in ultrapure water) in a final volume of 270 μL. The mixture was incubated for 30 min at 37 °C in a multiwell plate, and the absorbance was then read at 410 nm using a SpectraMax 340 multiwell plate reader (Molecular Devices), with one unit of activity being defined as an increase in absorbance of 0.001/min.

#### 4.4.4. L-Amino Acid Oxidase (LAAO) Activity

LAAO assay was assayed essentially as described elsewhere [89]. The standard assay mixture contained 90 μL of reaction buffer [250 mM L-methionine, 2 mM *o*-phenylenediamine (OPD), and 0.8 U/mL horseradish peroxidase in 50 mM Tris-HCl, pH 8.0] and 10 μL of venom (1 mg/mL, in ultrapure water) in a final volume of 100 μL. After incubation at 37 °C for 1 h, the reaction was stopped by adding 50 μL of 2 M H_2_SO_4_, and the resulting absorbance was read at 492 nm in a SpectraMax 340 multiwell plate reader (Molecular Devices). LAAO activity was estimated based on a standard curve of H_2_O_2_, with the results being expressed as μM of H_2_O_2_ produced/min/mg of venom. All samples were assayed in triplicate.

### 4.5. Coagulant Activity

For coagulant activity assays using human plasma, peripheral blood was collected from healthy adult volunteers and immediately centrifuged to obtain citrated platelet-poor plasma; these protocols were approved by the National Human Research Ethics Committee of the Brazilian Ministry of Health (Brasília, DF, Brazil; CAAE no. 43560620.0.0000.5493).

#### 4.5.1. Minimum Coagulant Dose

The minimum coagulant dose (MCD), defined as the minimum amount of venom capable of clotting a solution of fibrinogen or plasma in 60 s at 37 °C [90], was assayed using 50 µL of venom in different concentrations (50–500 µg/mL) and 50 µL of 25 mM CaCl_2_. This mixture was incubated for 120 s at 37 °C, and then 100 µL of human citrated plasma was added. The coagulation time was measured using a benchtop CA51 coagulation analyzer (Shenzhen Genius Electronics Co., Ltd., Shenzhen, China). All samples were assayed in triplicate.

#### 4.5.2. Prothrombin (PT) and Activated Partial Thromboplastin (aPTT) Clotting Times

The activated partial thromboplastin time (aPTT) and prothrombin time (PT) were determined using commercial kits with a Quick Timer Coagmaster 4.0 (Wama Diagnóstica Produtos para Laboratórios, São Carlos, SP, Brazil). For aPTT, the standard assay mixture contained 100 μL of rat citrated plasma, 100 μL of aPTT reagent, 100 μL of 0.02 M CaCl_2_, and 10 μL of venom (0.5 or 1.0 mg/mL, in ultrapure water) in a final volume of 310 μL; the reactions were initiated by adding CaCl_2_. For PT, the standard assay mixture contained 100 μL of rat citrated plasma, 200 μL of PT reagent, and 10 μL of venom (0.5 or 1.0 mg/mL, in ultrapure water) in a final volume of 310 μL. For neutralization assays, the venoms were preincubated with AV (AV:venom ratio of 1:1.5, *v*/*w*) for 20 min at 37 °C prior to assaying the residual activity on aPTT and PT.

#### 4.5.3. Thrombin-like Activity

The amidolytic activity of the venoms was assessed using a chromogenic substrate, S-2238 (Chromogenix^®^, Werfen, Barcelona, Spain), specific for thrombin, according to the manufacturer’s recommendations, with some modifications. For this assay, 5 μL of venom (1 mg/mL, in ultrapure water) was added to 10 μL of chromogenic substrate S-2238 (5.6 mM H-D-Phe-PIP-Arg-pNA⋅2HCl) and 90 μL of 50 mM Tris-HCl buffer, pH 7.4, containing 250 mM CaCl_2_, with the reaction being monitored at 405 nm for 20 min at 37 °C in a SpectraMax 340 multiwell plate reader (Molecular Devices). For the neutralization assays, the venoms were preincubated with antivenom (AV:venom ratio of 1:1.5, *v*/*w*) for 20 min at 37 °C prior to assaying the residual thrombin-like activity, as described above. The samples were assayed in triplicate.

#### 4.5.4. Fibrinogenolytic Activity

Venom fibrinogenolytic activity was assayed as described by Peichoto et al. (2007) [91]. Two hundred microliters of human fibrinogen (2 mg/mL, in ultrapure water) (Sigma-Aldrich Chemical Co., St. Louis, MO, USA) was incubated at 37 °C with 10 μg of venom samples. At various time intervals (5, 30, and 90 min), aliquots were withdrawn from the digestion mixture, mixed with denaturing solution (4% SDS, 20% glycerol, and 20% 2-mercaptoethanol), and reduced by boiling for 7 min prior to SDS-PAGE. Specific cleavage of fibrinogen by venom samples was determined by SDS-PAGE using 10% polyacrylamide gels. For the neutralization assays, venom samples (10 µg) were preincubated with AV (AV:venom ratio of 1:1.5, *v*/*w*) for 20 min at 37 °C, prior to assaying the residual fibrinogenolytic activity. Venom samples were also pre-incubated with 125 mM EDTA, an SVMP inhibitor, for 20 min at 37 °C. In both cases, the fibrinogenolytic activity was assayed as described above. After SDS-PAGE, the electrophoretic profiles were analyzed densitometrically using Gel Analyzer (version 23.1.1; developed by Istvan Lazar Jr. and Istvan Lazar Sr. and available at www.gelanalyzer.com; accessed on 5 July 2024).

### 4.6. Mouse Phrenic Nerve-Diaphragm (PND) Preparations

#### 4.6.1. Muscle Twitch-Tension Responses

Mouse hemidiaphragms and their phrenic nerves were dissected and mounted, as previously described [92]. The preparations were cleaned of connective tissue, and the diaphragm was divided into two triangular portions. The preparations were attached to hooks along their origin at the rib margin and mounted on custom-built tissue holders. The preparations were placed in 10 mL organ chambers under a resting tension of ~1 g in Tyrode physiological salt solution of the following composition (in mM): NaCl 137, KCl 2.7, CaCl_2_ 1.8, MgCl_2_ 0.49, NaH_2_PO_4_ 0.42, NaHCO_3_ 11.9, and glucose 11.1, pH 7.3–7.4, and aerated with carbogen (95% O_2_-5% CO_2_) at 37 °C. Muscle twitches evoked by supramaximal stimuli (0.2 Hz and 0.1 ms) delivered from a DataCapsule-Evo coupled to a Multiplexing Pulse Booster (Ugo Basile S.R.L., Gemonio, Varese, Italy) were recorded isometrically using DY1 force-displacement transducers (Ugo Basile). The muscle twitches were digitalized at a sampling interval of 10 ms using DataCapsule-Evo and recorded with LabScribe software, v.4 (Ugo Basile). After stabilization for 20 min, venom (3, 10, 30, and 100 μg/mL) was added to the chambers and left in contact with the preparations for up to 2 h or until complete blockade. For some experiments, the minimal venom concentration required to achieve a complete blockade was preincubated with AV at AV:venom ratios of 1:1.5 and 3:1.5 (*v*/*w*) for 20 min at 37 °C prior to testing for residual neuromuscular activity.

#### 4.6.2. Morphological Analysis

At the end of the myographical recordings, the diaphragm muscle was fixed in 10% formaldehyde overnight and then washed for 30 min in 0.1 M phosphate-buffered saline and 30 min in distilled water prior to storage in 70% ethanol overnight. The samples were dehydrated in graded ethanol (80%, 95%, and 100%), cleared in xylene (1:1 ethanol:xylene, 1:1 xylene:paraffin), and finally embedded in paraplast. Serial sections 5 μm thick (3–5 sections per diaphragm, separated from each other by 25 mm) were cut and mounted on plain glass slides for hematoxylin-eosin (HE) staining. The sections were analyzed in a blind fashion with the examiner being unaware of the treatments applied. The intensity of the alterations was assessed as discrete, moderate, or intense. The sections were examined using a Leica ICC50HD camera coupled to a Leica DM750 light microscope (Leica Microsystems, Wetzlar, Germany), and the images were captured and analyzed qualitatively using LAS 4.2 software (Leica Microsystems).

### 4.7. Statistical Analysis

The results were expressed as the mean ± SD, and statistical comparisons were performed with Student’s *t*-test or one-way ANOVA followed by the Tukey test, with *p* < 0.05 indicating significance. All data were analyzed using GraphPad Prism v.4.03 software (GraphPad Inc, San Diego, CA, EUA).

## 5. Conclusions

The findings of this investigation show that there are similarities and differences in the venom composition and biological activities among the three *C. durissus* ssp. examined, with important differences in the neutralizing capacity of crotalic AV produced by the Instituto Butantan. Specifically, CDRy venom showed greater proteolytic and LAAO activities than the other venoms, as well as differences in neuromuscular blocking activity and the ability to degrade the α-chain of fibrinogen. Crotalic AV effectively attenuated or inhibited most of the *C. d. ruruima* venom activities to an extent similar to that seen with *C. d. terrificus* venom. However, AV was less effective in inhibiting the α-chain fibrinogenolytic activity of *C. d. ruruima* venom. This observation suggests that further studies are necessary to better understand the neutralizing efficacy of crotalic AV produced by the Instituto Butantan against envenomation by different subspecies of *C. durissus* snakes in Brazil and beyond. Considering the geographical isolation of *C. d. ruruima*, our findings may have a bearing on the use of crotalic AV for treating rattlesnake bites in the northernmost region of Brazil, particularly since the AV currently produced is raised against venoms only from *C. d. collilineatus* and *C. d. terrificus* and may therefore not fully address the clinical manifestations of envenomation by *C. d. ruruima*.

## Figures and Tables

**Table 1:** Times for 50% and 90% neuromuscular blockade by *C. durissus* (CDT, CDRy, and CDRw) venoms in mouse PND preparations.

Venom (μg/mL)	50% Blockade (min)			90% Blockade (min)		
	CDT	CDRy	CDRw	CDT	CDRy	CDRw
3	NA	77 ± 4.5 ^O^	64 ± 4.9 ^O^	NA	108 ± 5.0 ^O^	102 ± 4.8 ^O^
10	79 ± 4.3 *	64 ± 4.0	51 ± 3.5 ^O^	106 ± 5.5 *	86 ± 4.5 ^O^	85 ± 4.0 ^O^
30	66 ± 4.8 *	48 ± 3.5 *	55 ± 4.4	95 ± 5.1 *	68 ± 3.4 *^O^	89 ± 5.1 ^λ^
100	48 ± 3.7 *	51 ± 4.2 *	50 ± 5.1 *	83 ± 4.6 *	72 ± 5.5 *	68 ± 4.2 *^O^

The values are the mean ± SD (*n* = 3). * *p* < 0.05 compared to times for 50% and 90% blockade reached with the lowest concentration (3 μg/mL) of each venom. ^O^ *p* < 0.05 compared to the corresponding time for blockade by CDT venom. ^λ^ *p* < 0.05 compared to the corresponding time for blockade by CDRy venom. NA: not applicable—the level of blockade was not reached during a 2 h incubation.

**Table 2:** Times for 50% neuromuscular blockade by *C. d. terrificus* (CDT) and *C. d. ruruima* (CDRy and CDRw) venoms (10 μg/mL) pretreated with anti-*Crotalus* antivenom and assayed in mouse PND preparations.

Treatments	50% Blockade (min)		
	CDT	CDRy	CDRw
V (10 μg/mL)	79 ± 4.3	64 ± 4.0	51 ± 3.5 ^#^
V (10 μg/mL) + Antivenom (1:1.5)	76 ± 7.1	88 ± 5.9 *	102 ± 8.3 *
V (10 μg/mL) + Antivenom (3:1.5)	97 ± 5.7 *	NA	NA

Values are the mean ± SD (*n* = 3). * *p* < 0.05 compared to the time for 50% blockade by venom alone. ^#^ *p* < 0.05 compared to CDT. NA: not applicable—this level of blockade was not reached during a 2 h incubation.

## Data Availability

Data will be made available on request.
